# Prognostic value of LODDS in late-onset gastric adenocarcinoma: analysis of SEER, TCGA, and external multicenter cohorts

**DOI:** 10.3389/fonc.2025.1619504

**Published:** 2025-10-06

**Authors:** Hengbo Xia, Yong Qian, Qingqing Pang, Aman Xu, Jie Hu

**Affiliations:** ^1^ Department of General Surgery, The First Affiliated Hospital of Anhui Medical University, Hefei, Anhui, China; ^2^ Department of Respiratory and Critical Care Medicine, The First Affiliated Hospital of Anhui Medical University, Hefei, Anhui, China; ^3^ Department of Gastrointestinal Surgery, Shuguang Hospital Affiliated to Shanghai University of Traditional Chinese Medicine, Shanghai, China

**Keywords:** late-onset gastric adenocarcinoma, LODDS, prognostic model, cause-specific survival, overall survival

## Abstract

**Introduction:**

Accurate survival prediction is crucial for optimizing individualized treatment and follow-up in patients with late-onset gastric adenocarcinoma (LOGA). Traditional lymph node staging systems such as N-stage, positive lymph node (PLN), and lymph node ratio (LNR) have limitations in predictive accuracy, especially in cases with inadequate lymph node dissection. The log odds of positive lymph nodes (LODDS), a novel nodal staging metric that incorporates both positive and negative lymph nodes through a log-transformed ratio, has shown potential advantages by providing a more stable and refined assessment of nodal involvement.

**Materials and methods:**

This study included 10,361 LOGA patients from the SEER database, 135 from TCGA, and 252 from two medical centers. A novel prognostic model was constructed based on a training cohort from SEER and validated using internal (SEER testing set) and external (TCGA and hospital datasets) cohorts. The model incorporated age, gender, grade, size, chemotherapy, and LODDS. Four staging systems (TNM-stage, PLN-stage, LNR-stage, LODDS-stage) were compared using the Akaike Information Criterion (AIC), Concordance Index (C-index) and time-dependent Area Under the Curve (AUC). LODDS-stage model, the most effective model, was used to build nomograms for overall survival (OS) and cause-specific survival (CSS). Model performance was evaluated using calibration curves, Decision Curve Analysis (DCA), and Kaplan-Meier analysis.

**Results:**

Univariate and multivariate Cox regression identified age, gender, grade, tumor size, chemotherapy, and LODDS-stage as independent prognostic factors. Among the four models, the LODDS-based model showed the highest discrimination and best calibration for predicting OS and CSS at 1, 3, 5, and 10 years. Nomograms incorporating these variables exhibited excellent predictive accuracy in both internal and external cohorts. Survival risk classification based on model scores effectively stratified patients into high- and low-risk groups, with significantly different survival outcomes across all datasets (p < 0.05).

**Conclusions:**

The LODDS-based prognostic model outperformed traditional nodal staging systems in survival prediction for LOGA patients. This model showed high accuracy and consistent performance across different datasets, indicating its potential to support personalized treatment and long-term follow-up strategies for elderly patients with gastric cancer.

## Introduction

Gastric cancer is a major health issue worldwide. According to GLOBOCAN 2022, gastric cancer ranks fifth in both global incidence and cancer-related mortality (1). Gastric adenocarcinoma, the predominant histological subtype, comprises the majority of these cases (2). As the population ages, the prevalence of late-onset gastric adenocarcinoma (LOGA), generally defined as diagnosis at 50 years of age or older, has been steadily increasing (3). Recent studies show that LOGA is different from early-onset gastric cancer in symptoms, molecular features, and treatment response (4). These differences show the need for better models to predict outcomes and guide treatment for this group of patients.

The American Joint Committee on Cancer (AJCC) TNM staging system (5) is widely adopted for prognostic evaluation in gastric cancer. However, the N stage, which is only based on the number of metastatic lymph nodes, does not take into account the total number of examined lymph nodes (ELNs) (6). This may lead to incorrect staging, especially if not enough lymph nodes are removed during surgery. To solve this problem, alternative lymph node-based indicators such as positive lymph node (PLN) (7), lymph node ratio (LNR) (8) and log odds of positive lymph nodes (LODDS) (9) have been suggested. While LNR incorporates both metastatic and examined lymph nodes, it is less accurate when the ratio is 0 or 1, limiting its utility in some cases (10).

LODDS, calculated as the log ratio between the number of positive and negative lymph nodes, offers a more comprehensive and stable method for assessing lymph node involvement (11). By integrating both positive and negative lymph node information, LODDS can reduce bias from differences in surgery or Pathological examination. Several studies in solid tumors (9, 12–14), including gastric cancer, have shown that LODDS better than PLN and LNR in predicting survival. Nevertheless, few research has specifically investigated the prognostic significance of LODDS in patients with LOGA, and its generalizability remains uncertain due to a lack of large-scale validation.

The present study utilized population-based data from the SEER and TCGA databases, complemented by multicenter external validation cohorts, to evaluate the prognostic value of LODDS in LOGA. By comparing LODDS with N-stage, PLN and LNR, we aim to determine its relative predictive accuracy and clinical applicability. Furthermore, we developed a nomogram incorporating LODDS-stage and other relevant variables to facilitate personalized risk assessment and guide clinical decision-making in this aging patient population.

## Materials and methods

### Data source

Clinical and pathological data of patients with LOGA who underwent radical gastrectomy with lymphadenectomy were collected from four sources: (1) the SEER database, (2) the TCGA database, (3) The First Affiliated Hospital of Anhui Medical University, (4) The Fourth Affiliated Hospital of Anhui Medical University.

**Figure 1:**
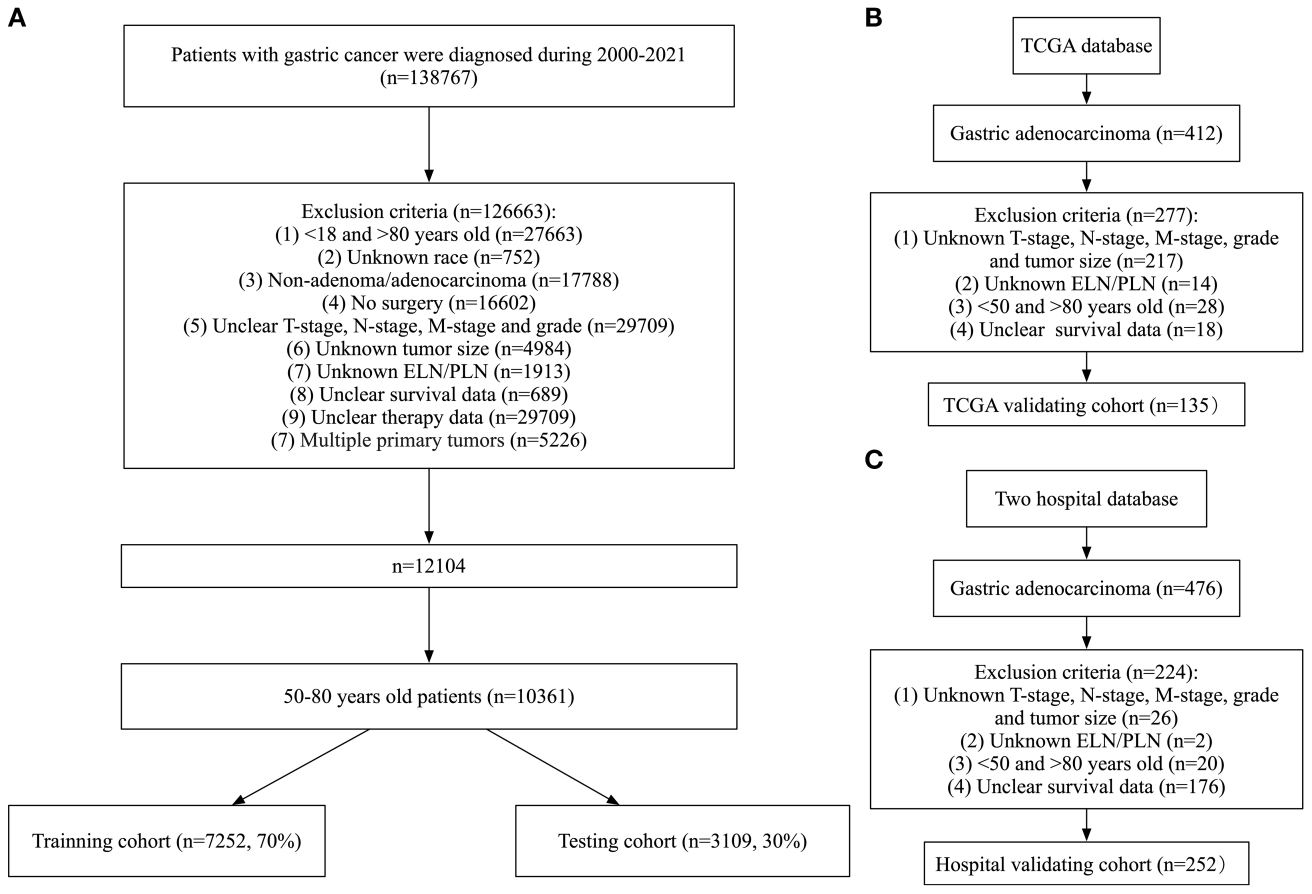
Flowchart of data collection and grouping for patients with LOGA. **(A)** SEER cohort; **(B)** TCGA validating cohort; **(C)** Hospital validating cohort.

Patients were excluded based on the following criteria: (1) age <50 or >80 years at diagnosis; (2) incomplete clinical or pathological data; (3) presence of multiple primary tumors; (4) missing lymph node dissection data. A total of 10,361 patients from SEER, 135 from TCGA, 99 from The First Affiliated Hospital, and 153 from The Fourth Affiliated Hospital were included in the present study (**Figure 1**). Patients from the SEER database were randomly divided into a training set(n=7252, 70%) and a testing set(n=3109, 30%). The training set was used to develop the prognostic prediction models. Data from the testing set and the two hospitals were used as independent external validation groups for examination of the prognostic prediction model.

Data from SEER and TCGA were exempt from ethical approval. This retrospective study was conducted using previously recorded clinical data, without involving any personally identifiable information. The study protocol was reviewed and approved by the Ethics Committee of The First Affiliated Hospital of Anhui Medical University (J 2025-03-72) and conducted in accordance with the Declaration of Helsinki. Given the non-interventional and anonymized nature of the study, the requirement for informed consent was waived by the Ethics Committee.

### Selection and definition of clinicopathological parameters

The following clinicopathological variables were included in this study: age, gender, primary site, grade, T-stage, N-stage, M-stage, TNM-stage, radiation, chemotherapy, size, ELN, PLN, LNR, LODDS, overall survival (OS), cause-specific survival (CSS), survival months. LNR was defined as the ratio of PLN to ELN. LODDS was calculated using the formula: log[(PLN + 0.5)/(ELN - PLN + 0.5)]. Overall survival (OS) and cause-specific survival (CSS) were the primary and secondary endpoints, respectively, and were derived from the SEER variables “COD to site record” and “SEER cause-specific death classification.”

### Processing of data

Age and tumor size were treated as continuous variables. The primary site was classified into nine subgroups: cardia, fundus of stomach, body of stomach, gastric antrum, pylorus, lesser curvature of stomach, greater curvature of stomach, overlapping lesion of stomach, stomach(NOS). Grade was divided into four subgroups: well, moderate, poor, undifferentiated/anaplastic. Based on the 8th edition of the AJCC staging system, T stage was divided into five categories: T1, T2, T3, T4a and T4b. N stage was divided into five categories: N0, N1, N2, N3a and N3b. M stage was divided into two categories: M0 and M1. TNM stage was divided into eight categories: IA, IB, IIA, IIB, IIIA, IIIB, IIIC, IV. Radiation and chemotherapy were recorded as None/Unknown or YES.

The optimal cut-off values for LODDS, LNR, and PLN were determined using X-tile software (version 3.6.1). Based on these thresholds, patients were divided into three groups for each metric:

LODDS: LODDS1 (−2.26 to −0.92), LODDS2 (−0.92 to 0.16), LODDS3 (0.16 to 2.00);
LNR: LNR1 (0 to 0.0769), LNR2 (0.0769 to 0.594), LNR3 (0.594 to 1);
PLN: PLN1 (0), PLN2 (1–6), PLN3 (≥7).

### Construction of new staging system

**Figure 2:**
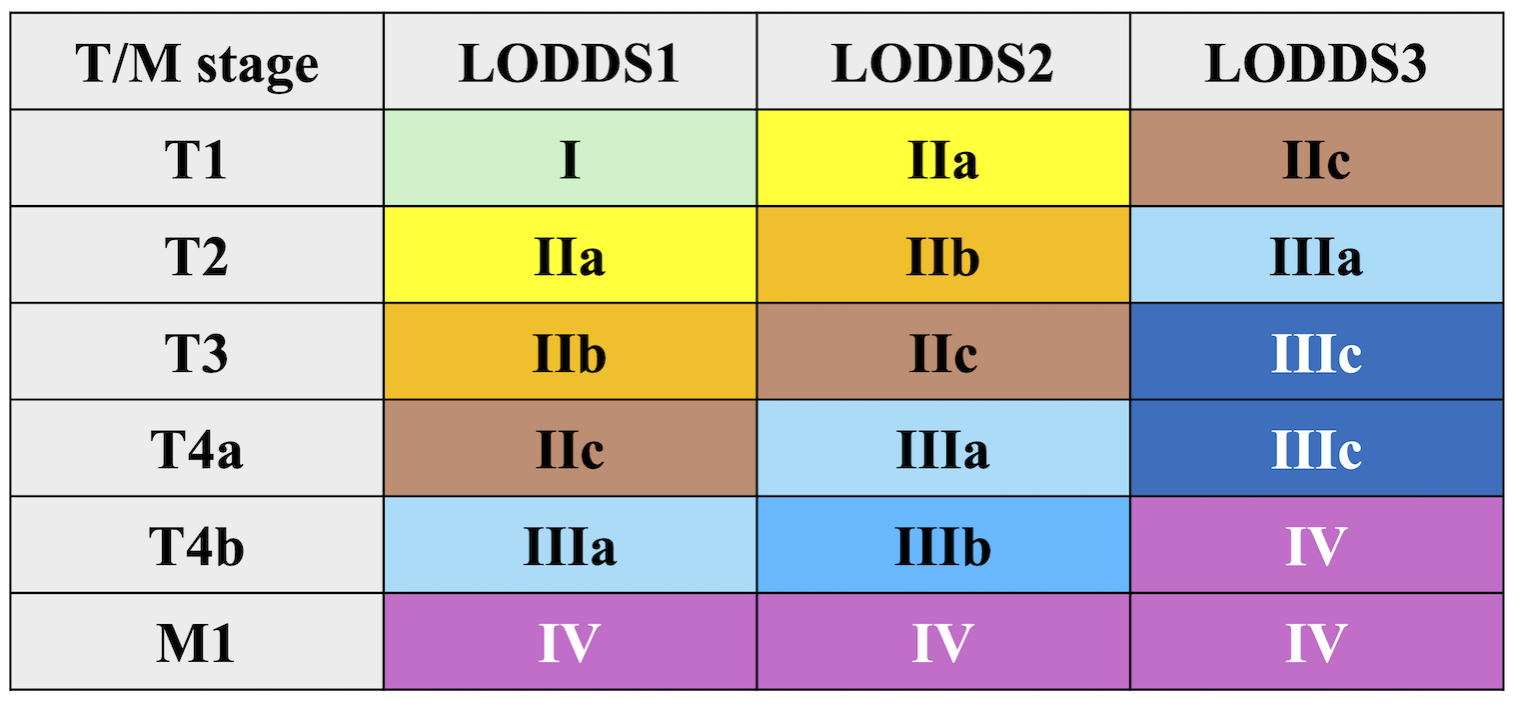
A novel LODDS stage classification.

A new staging system was developed by integrating LODDS with T and M stages, following the structure of the AJCC 8th edition. This model initially included 18 subgroups, which were subsequently combined into 9 final stages (**Figure 2**):

Stage I: T1LODDS1M0
Stage IIa: T2LODDS1M0, T1LODDS2M0
Stage IIb: T3LODDS1M0, T2LODDS2M0
Stage IIc: T4aLODDS1M0, T3LODDS2M0, T1LODDS3M0
Stage IIIa: T4bLODDS1M0, T4aLODDS2M0, T2LODDS3M0
Stage IIIb: T4bLODDS2M0
Stage IIIc: T3–T4aLODDS3M0
Stage IV: T4bLODDS3M0, M1

Similar grouping methods were applied to PLN and LNR-based models.

### Construction and comparison of four staging models

Cox regression analysis was performed using the training set. Variables with statistical significance in univariate analysis were included in multivariate analysis. Four prognostic models were constructed based on TNM, PLN, LNR, and LODDS classifications. Model performance was compared using the Akaike Information Criterion (AIC), Concordance Index (C-index), and Area Under the Curve (AUC). AIC is a statistical measure that aims to optimize the balance between model fit and complexity, penalizing excessive parameters to prevent overfitting. The C-index evaluates a model’s ability to discriminate risk rankings, particularly in survival analysis, where it measures concordance between predicted and observed event sequences. The AUC is a metric that assesses the performance of a binary classification model by quantifying the separation between classes across all possible decision thresholds. It is notable for its resilience to class imbalance. Collectively, these metrics comprehensively address model parsimony, predictive consistency, and discriminative power across a range of applications.

### Construction and validation of nomograms

Based on the training set, nomograms were developed to predict OS and CSS by integrating the most predictive variables. The evaluation of the applicability of the nomograms was performed using the C-index, time-dependent AUC, and calibration curves in the training and testing sets. Decision Curve Analysis (DCA) was performed to assess clinical utility by calculating net benefit across different threshold probabilities.

### Classification of survival risk on basis of prognostic prediction models

Patients were categorized into high- and low-risk groups according to the calculated survival risk scores derived from the nomogram. Survival differences between the two groups were analyzed using Kaplan-Meier curves and the log-rank test.

### Statistical analysis

Categorical variables were reported as frequencies (n) and percentages (%). Continuous variables with normal distribution were expressed as mean ± standard deviation (SD) and compared using independent-sample t-tests. Non-normally distributed variables were presented as median and interquartile range (IQR), and compared using the Mann-Whitney U test. All statistical tests were two-sided, and a p value <0.05 was considered statistically significant. Given that all the analyses were based on predefined hypotheses and clinically relevant variables rather than exploratory multiple testing, no formal adjustment for multiple comparisons was applied. All the statistical analyses in this study were conducted using R software (version 4.4.2) and GraphPad Prism (version 10.2.3).

## Results

### Characteristics of patients with LOGA from SEER database

A total of 10,361 patients diagnosed with LOGA extracted from the SEER database were randomly divided into training and testing sets. As presented in **Table 1**, no statistically significant differences in clinicopathological parameters were observed between the two groups (p>0.05).

**Table 1:** Clinical and pathologic characteristics of patients with LOGA in two cohorts.

Characteristics	Level	Overall	Training set	Testing set	P
		n=10361	n=7252	n=3109	
Age (median [IQR])		67.00 [59.00, 73.00]	67.00 [59.00, 73.00]	66.00 [59.00, 73.00]	0.116
Size (median [IQR])		4.20 [2.50, 6.50]	4.20 [2.50, 6.50]	4.20 [2.50, 6.50]	0.875
Survival months (median [IQR])		33.00 [12.00, 98.00]	33.00 [12.00, 99.00]	31.00 [12.00, 96.00]	0.221
Gender (%)	Female	3568 (34.4)	2460 (33.9)	1108 (35.6)	0.096
	Male	6793 (65.6)	4792 (66.1)	2001 (64.4)	
Primary site (%)	Cardia	2975 (28.7)	2097 (28.9)	878 (28.2)	0.571
	Fundus of stomach	326 (3.1)	236 (3.3)	90 (2.9)	
	Body of stomach	966 (9.3)	655 (9.0)	311 (10.0)	
	Gastric antrum	2648 (25.6)	1874 (25.8)	774 (24.9)	
	Pylorus	424 (4.1)	299 (4.1)	125 (4.0)	
	Lesser curvature of stomach	1189 (11.5)	821 (11.3)	368 (11.8)	
	Greater curvature of stomach	484 (4.7)	342 (4.7)	142 (4.6)	
	Overlapping lesion of stomach	708 (6.8)	495 (6.8)	213 (6.9)	
	Stomach, NOS	641 (6.2)	433 (6.0)	208 (6.7)	
Grade (%)	Well differentiated	482 (4.7)	325 (4.5)	157 (5.0)	0.133
	Moderately differentiated	2874 (27.7)	2049 (28.3)	825 (26.5)	
	Poorly differentiated	6743 (65.1)	4704 (64.9)	2039 (65.6)	
	Undifferentiated/anaplastic	262 (2.5)	174 (2.4)	88 (2.8)	
T stage (%)	T1	2112 (20.4)	1448 (20.0)	664 (21.4)	0.594
	T2	3250 (31.4)	2280 (31.4)	970 (31.2)	
	T3	3279 (31.6)	2316 (31.9)	963 (31.0)	
	T4a	1114 (10.8)	782 (10.8)	332 (10.7)	
	T4b	606 (5.8)	426 (5.9)	180 (5.8)	
N stage (%)	N0	4029 (38.9)	2838 (39.1)	1191 (38.3)	0.8
	N1	1895 (18.3)	1317 (18.2)	578 (18.6)	
	N2	1907 (18.4)	1332 (18.4)	575 (18.5)	
	N3a	1738 (16.8)	1202 (16.6)	536 (17.2)	
	N3b	792 (7.6)	563 (7.8)	229 (7.4)	
M stage (%)	M0	9295 (89.7)	6530 (90.0)	2765 (88.9)	0.095
	M1	1066 (10.3)	722 (10.0)	344 (11.1)	
Stage (%)	IA	1663 (16.1)	1146 (15.8)	517 (16.6)	0.32
	IB	1422 (13.7)	1000 (13.8)	422 (13.6)	
	IIA	1610 (15.5)	1161 (16.0)	449 (14.4)	
	IIB	1336 (12.9)	925 (12.8)	411 (13.2)	
	IIIA	1515 (14.6)	1065 (14.7)	450 (14.5)	
	IIIB	1162 (11.2)	813 (11.2)	349 (11.2)	
	IIIC	587 (5.7)	420 (5.8)	167 (5.4)	
	IV	1066 (10.3)	722 (10.0)	344 (11.1)	
Radiation (%)	None/Unknown	6556 (63.3)	4574 (63.1)	1982 (63.8)	0.526
	YES	3805 (36.7)	2678 (36.9)	1127 (36.2)	
Chemotherapy (%)	None/Unknown	4647 (44.9)	3219 (44.4)	1428 (45.9)	0.154
	YES	5714 (55.1)	4033 (55.6)	1681 (54.1)	
PLN (%)	0	4029 (38.9)	2838 (39.1)	1191 (38.3)	0.73
	1-6	3802 (36.7)	2649 (36.5)	1153 (37.1)	
	≥7	2530 (24.4)	1765 (24.3)	765 (24.6)	
LNR (%)	0 - 0.0769	4683 (45.2)	3290 (45.4)	1393 (44.8)	0.87
	0.0769 - 0.594	3790 (36.6)	2644 (36.5)	1146 (36.9)	
	0.594 - 1	1888 (18.2)	1318 (18.2)	570 (18.3)	
LODDS (%)	−2.26 - −0.92	4438 (42.8)	3130 (43.2)	1308 (42.1)	0.573
	−0.92 - 0.16	4068 (39.3)	2827 (39.0)	1241 (39.9)	
	0.16 - 2.00	1855 (17.9)	1295 (17.9)	560 (18.0)	
PLN stage (%)	I	1663 (16.1)	1146 (15.8)	517 (16.6)	0.571
	IIa	1537 (14.8)	1077 (14.9)	460 (14.8)	
	IIb	2085 (20.1)	1494 (20.6)	591 (19.0)	
	Iic	1409 (13.6)	981 (13.5)	428 (13.8)	
	IIIa	999 (9.6)	704 (9.7)	295 (9.5)	
	IIIb	241 (2.3)	166 (2.3)	75 (2.4)	
	IIIc	1182 (11.4)	833 (11.5)	349 (11.2)	
	IV	1245 (12.0)	851 (11.7)	394 (12.7)	
LNR stage (%)	I	1770 (17.1)	1215 (16.8)	555 (17.9)	0.226
	IIa	1654 (16.0)	1176 (16.2)	478 (15.4)	
	IIb	2271 (21.9)	1616 (22.3)	655 (21.1)	
	Iic	1497 (14.4)	1029 (14.2)	468 (15.1)	
	IIIa	917 (8.9)	659 (9.1)	258 (8.3)	
	IIIb	235 (2.3)	168 (2.3)	67 (2.2)	
	IIIc	798 (7.7)	561 (7.7)	237 (7.6)	
	IV	1219 (11.8)	828 (11.4)	391 (12.6)	
LODDS stage (%)	I	1604 (15.5)	1111 (15.3)	493 (15.9)	0.503
	IIa	1773 (17.1)	1250 (17.2)	523 (16.8)	
	IIb	2311 (22.3)	1638 (22.6)	673 (21.6)	
	Iic	1526 (14.7)	1053 (14.5)	473 (15.2)	
	IIIa	900 (8.7)	645 (8.9)	255 (8.2)	
	IIIb	250 (2.4)	179 (2.5)	71 (2.3)	
	IIIc	782 (7.5)	551 (7.6)	231 (7.4)	
	IV	1215 (11.7)	825 (11.4)	390 (12.5)	
OS (%)	Alive	2947 (28.4)	2073 (28.6)	874 (28.1)	0.641
	Dead	7414 (71.6)	5179 (71.4)	2235 (71.9)	
CSS (%)	Alive	4547 (43.9)	3186 (43.9)	1361 (43.8)	0.9
	Dead	5814 (56.1)	4066 (56.1)	1748 (56.2)	

LOGA, late-onset gastric adenocarcinoma; IQR, interquartile range; LNR, lymph node ratio; LODDS, log odds of positive lymph node; PLN, positive lymph node; OS, overall survival; CSS, cause specific survival.

### Univariate and multivariate cox regression analysis for OS and CSS

Firstly, 18 clinicopathologic variables were included in a univariate COX regression analysis, 16 of which were significantly associated with OS and 17 with CSS (**Table 2**). Subsequently, to address potential multicollinearity, certain variables were consolidated. In the analysis of OS, age, gender, grade, size and chemotherapy were included in the multivariate COX regression analysis in combination with one of TNM-stage, LNR-stage, PLN-stage and LODDS-stage, respectively. In the analysis of CSS, age, gender, grade, size, radiation and chemotherapy were included in the multifactorial COX regression analysis in combination with one of TNM-stage, LNR-stage, PLN-stage and LODDS-stage, respectively. The results showed that all included variables were identified as independent prognostic factors for OS (**Table 3**), and all but radiation were independent factors for CSS (**Table 4**). In conclusion, it’s consistent on both OS and CSS that age, gender, grade, size and chemotherapy in combination with one of TNM-stage, LNR-stage, PLN-stage and LODDS-stage were independent influence factors.

**Table 2:** Univariate cox regression analyses for predicting OS and CSS in the training cohort.

Characteristics	OS		CSS	
	HR (95% CI)	P-value	HR (95% CI)	P-value
Age	1.020 (1.017-1.023)	<0.001*	1.009 (1.005-1.012)	<0.001*
Gender	1.092 (1.031-1.158)	0.003*	1.080 (1.011-1.153)	0.022*
Primary site	1.006 (0.995-1.018)	0.264	1.002 (0.989-1.014)	0.776
Grade	1.301 (1.243-1.362)	<0.001*	1.503 (1.424-1.587)	<0.001*
T stage	1.436 (1.403-1.470)	<0.001*	1.557 (1.517-1.599)	<0.001*
N stage	1.470 (1.441-1.500)	<0.001*	1.597 (1.562-1.633)	<0.001*
M stage	2.864 (2.636-3.110)	<0.001*	3.335 (3.060-3.636)	<0.001*
Stage	1.317 (1.300-1.333)	<0.001*	1.406 (1.386-1.426)	<0.001*
Radiation	0.998 (0.943-1.056)	0.943	1.088 (1.022-1.159)	0.008*
Chemotherapy	1.130 (1.069-1.194)	<0.001*	1.338 (1.256-1.426)	<0.001*
Size	1.103 (1.094-1.112)	<0.001*	1.125 (1.115-1.134)	<0.001*
PLN stage	1.288 (1.272-1.303)	<0.001*	1.362 (1.344-1.380)	<0.001*
LNR stage	1.313 (1.298-1.329)	<0.001*	1.381 (1.363-1.400)	<0.001*
LODDS stage	1.317 (1.301-1.333)	<0.001*	1.384 (1.366-1.403)	<0.001*

OS, overall survival; CSS, cause specific survival; HR, hazard ratio; CI, confidence interval; PLN, positive lymph node. LNR, lymph node ratio; LODDS, log odds of positive lymph node.

* means statistically significant.

**Table 3:** Multivariate cox regression analyses for predicting OS in the training cohort.

Characteristics	TNM-stage		PLN-stage		LNR-stage		LODDS-stage	
	HR (95% CI)	P-value	HR (95% CI)	P-value	HR (95% CI)	P-value	HR (95% CI)	P-value
Age	1.026(1.023-1.030)	<0.001*	1.027(1.023-1.030)	<0.001*	1.026(1.022-1.029)	<0.001*	1.026(1.022-1.029)	<0.001*
Gender	1.144(1.079-1.213)	<0.001*	1.154(1.088-1.224)	<0.001*	1.141(1.076-1.210)	<0.001*	1.142(1.077-1.211)	<0.001*
grade	1.108(1.056-1.162)	<0.001*	1.123(1.071-1.178)	<0.001*	1.128(1.075-1.183)	<0.001*	1.129(1.077-1.185)	<0.001*
Chemotherapy	0.736(0.694-0.781)	<0.001*	0.754(0.711-0.801)	<0.001*	0.785(0.740-0.832)	<0.001*	0.791(0.745-0.839)	<0.001*
Size	1.032(1.022-1.042)	<0.001*	1.036(1.026-1.046)	<0.001*	1.036(1.026-1.046)	<0.001*	1.037(1.027-1.047)	<0.001*
TNM-stage	1.328(1.310-1.347)	<0.001*	/	/	/	/	/	/
PLN-stage	/	/	1.294(1.277-1.312)	<0.001*	/	/	/	/
LNR-stage	/	/	/	/	1.311(1.294-1.328)	<0.001*	/	/
LODDS-stage	/	/	/	/	/	/	1.313(1.296-1.331)	<0.001*

OS, overall survival; HR, hazard ratio; CI, confidence interval; PLN, positive lymph node; LNR, lymph node ratio; LODDS, log odds of positive lymph node.

* means statistically significant.

**Table 4:** Multivariate cox regression analyses for predicting CSS in the training cohort.

Characteristics	TNM-stage		PLN-stage		LNR-stage		LODDS-stage	
	HR (95% CI)	P-value	HR (95% CI)	P-value	HR (95% CI)	P-value	HR (95% CI)	P-value
Age	1.017(1.013-1.021)	<0.001*	1.018(1.014-1.021)	<0.001*	1.017(1.013-1.021)	<0.001*	1.017(1.013-1.021)	<0.001*
Gender	1.126(1.054-1.202)	<0.001*	1.138(1.065-1.215)	<0.001*	1.121(1.050-1.198)	<0.001*	1.124(1.052-1.200)	<0.001*
Grade	1.207(1.141-1.278)	<0.001*	1.228(1.161-1.300)	<0.001*	1.237(1.170-1.309)	<0.001*	1.239(1.171-1.311)	<0.001*
Radiation	0.978(0.908-1.052)	0.543	0.965(0.896-1.039)	0.340	1.007(0.935-1.085)	0.853	1.007(0.935-1.085)	0.847
Chemotherapy	0.802(0.742-0.866)	<0.001*	0.827(0.765-0.895)	<0.001*	0.849(0.785-0.918)	<0.001*	0.856(0.792-0.926)	<0.001*
Size	1.039(1.028-1.050)	<0.001*	1.043(1.033-1.054)	<0.001*	1.044(1.033-1.055)	<0.001*	1.045(1.034-1.056)	<0.001*
TNM-stage	1.392(1.370-1.415)	<0.001*	/	/	/	/	/	/
PLN-stage	/	/	1.345(1.325-1.365)	<0.001*	/	/	/	/
LNR-stage	/	/	/	/	1.358(1.338-1.378)	<0.001*	/	/
LODDS-stage	/	/	/	/	/	/	1.360(1.340-1.380)	<0.001*

CSS, cause-specific survival; HR, hazard ratio; CI, confidence interval; PLN, positive lymph node; LNR, lymph node ratio; LODDS, log odds of positive lymph node.

* means statistically significant.

### Comparison of four staging-based prognostic prediction models

AIC, C-index, and AUC were used to compare the performance of the four staging-based prognostic prediction models in the training group. In the analysis of OS, LODDS-stage model demonstrated the lowest AIC value, the highest C-index and time-dependent AUC values at 1, 3, 5, and 10 years in comparison with the other three models. In the analysis of CSS, LODDS-stage model outperformed the other three staging systems in terms of C-index and time-dependent AUC across all evaluated time points (**Table 5**), indicating superior overall predictive performance.

**Table 5:** Prognostic efficiency of different lymph node status indicators in the training cohort.

Endpoint	Filtered model	C-index	AIC	AUC			
				1-year	3-year	5-year	10-year
OS	TNM-stage	0.704	83678.30	0.752	0.778	0.783	0.773
	PLN-stage	0.699	83764.04	0.748	0.772	0.775	0.767
	LNR-stage	0.705	83646.14	0.757	0.779	0.782	0.776
	LODDS-stage	0.706	83636.19	0.758	0.780	0.784	0.777
CSS	TNM-stage	0.729	66068.26	0.770	0.800	0.806	0.805
	PLN-stage	0.725	66184.81	0.767	0.795	0.800	0.799
	LNR-stage	0.730	66096.57	0.774	0.801	0.806	0.805
	LODDS-stage	0.730	66094.94	0.775	0.803	0.807	0.805

OS, overall survival; CSS, cause-specific survival; AIC, Akaike information criterion; AUC, the area under the curve; C-index, concordance index; PLN, positive lymph node; LNR, lymph node ratio; LODDS, log odds of positive lymph node.

### Construction and validation of nomograms

**Figure 3:**
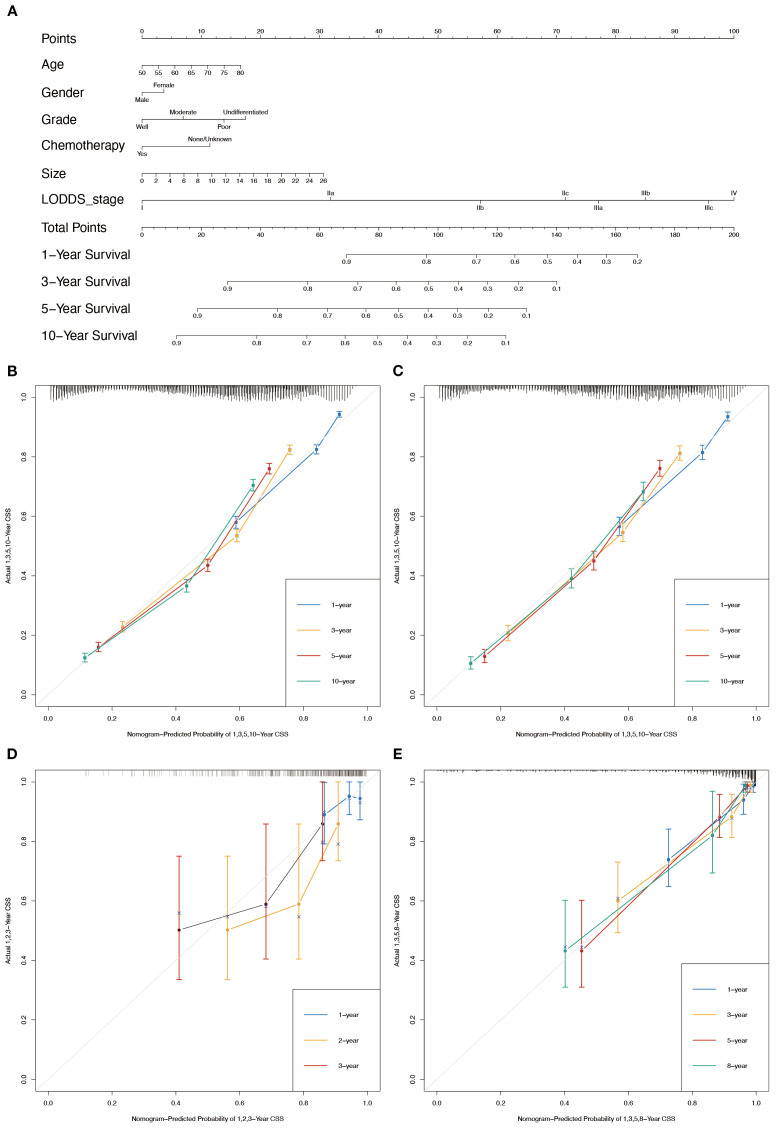
Nomogram for the OS of patients with LOGA. **(A)** 1-, 3-, 5-, and 10-years OS predictions based on the nomogram. Calibration plots for 1-, 3-, 5- and 10-years in training set **(B)** and testing set **(C)**. Calibration plots for 1-, 2-, and 3-years in TCGA validation set **(D)**. Calibration plots for 1-, 3-, 5-, and 8-years in hospital validation set **(E)**.

**Figure 4:**
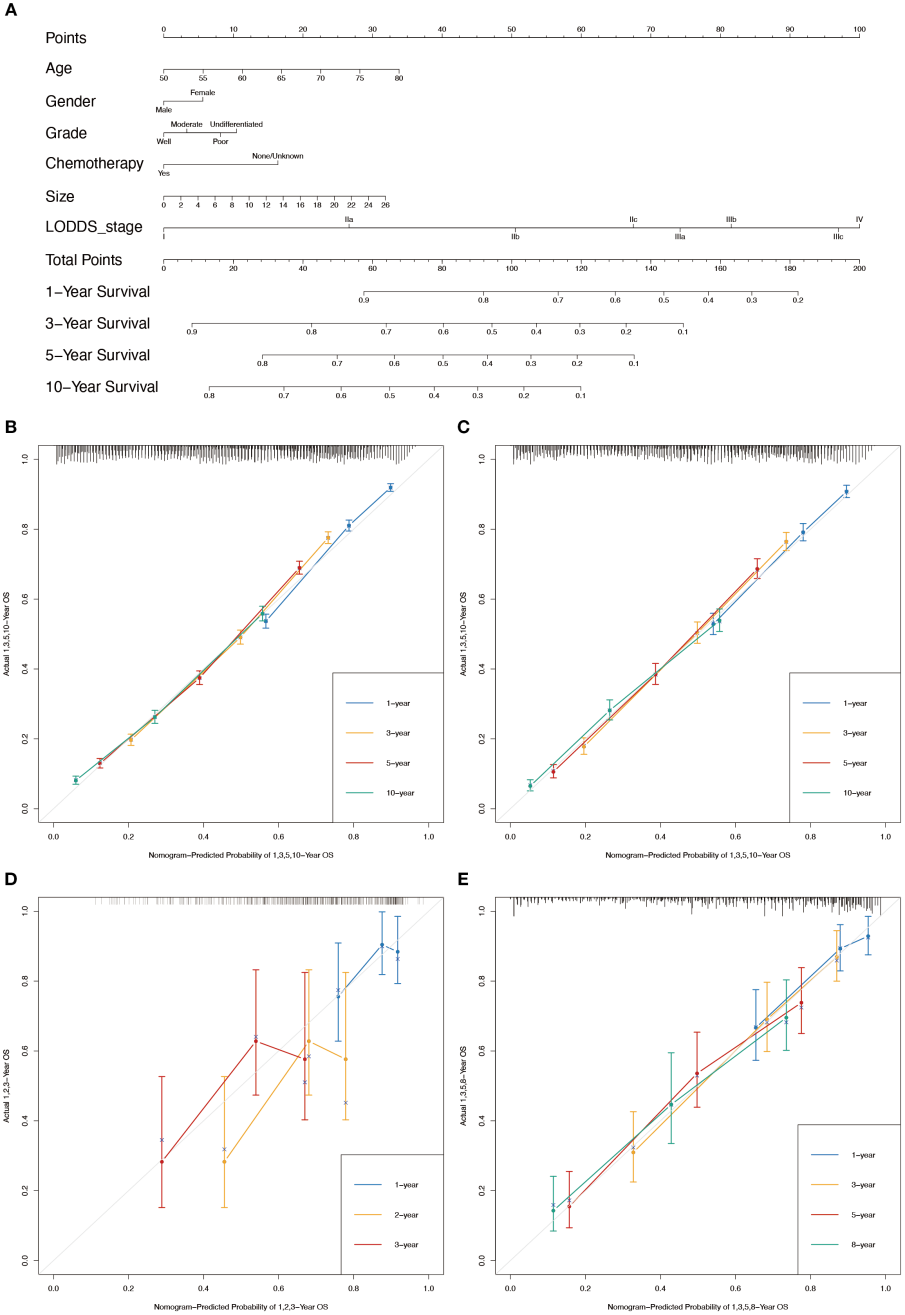
Nomogram for the CSS of patients with LOGA. **(A)** 1-, 3-, 5-, and 10-years CSS predictions based on the nomogram. Calibration plots for 1-, 3-, 5- and 10-years in training set **(B)** and testing set **(C)**. Calibration plots for 1-, 2-, and 3-years in TCGA validation set **(D)**. Calibration plots for 1-, 3-, 5-, and 8-years in hospital validation set **(E)**.

In conjunction with the above studies, the prognostic prediction model integrating LODDS-stage was found to be more comprehensive and accurate. Therefore, this model was utilized to construct the nomograms for predicting 1-, 3-, 5-, and 10-year OS (**Figure 3A**) and CSS (**Figure 4A**) in LOGA patients. Subsequently internal validation based on the training set and external validation based on the testing set, the TCGA dataset, and the hospital dataset, the calibration curves demonstrated excellent agreement between predicted and observed survival probabilities (**Figures 3B-D**; **Figures 4B-D**).

**Figure 5:**
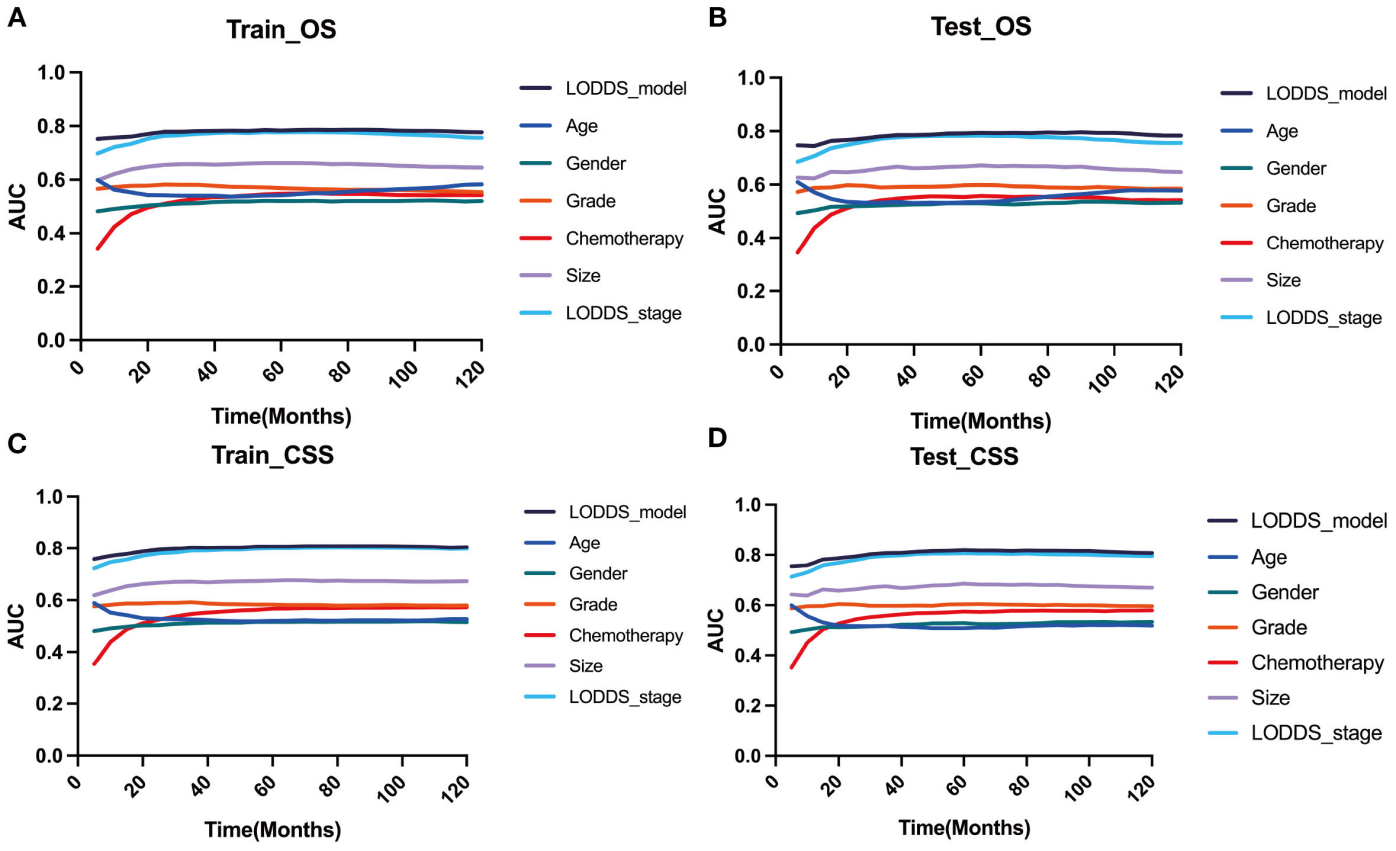
Comparison of the time‐dependent AUC of the LODDS stage model with a single characteristic for OS in the training **(A)** and testing **(B)** cohorts. Comparison of the time‐dependent AUC of the LODDS stage model with a single characteristic for CSS in the training **(C)** and testing **(D)** cohorts.

**Figure 6:**
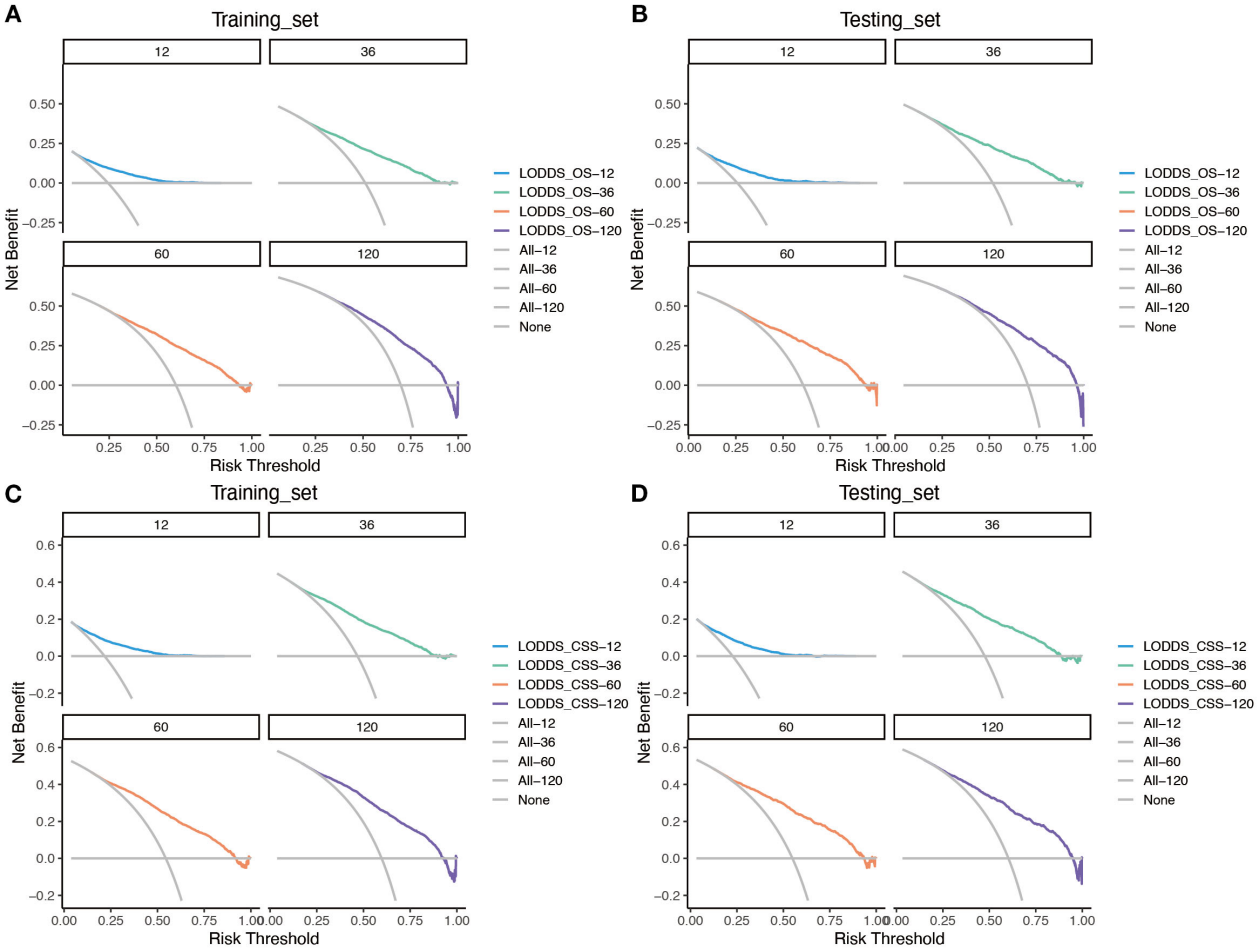
Evaluation of the nomogram for the OS and CSS of patients with LOGA using time-dependent AUC. The time‐dependent AUC for the OS of patients in the training **(A)** and testing **(B)** cohorts. The time‐dependent AUC for the CSS of patients in the training **(C)** and testing **(D)** cohorts.

**Figure 7:**
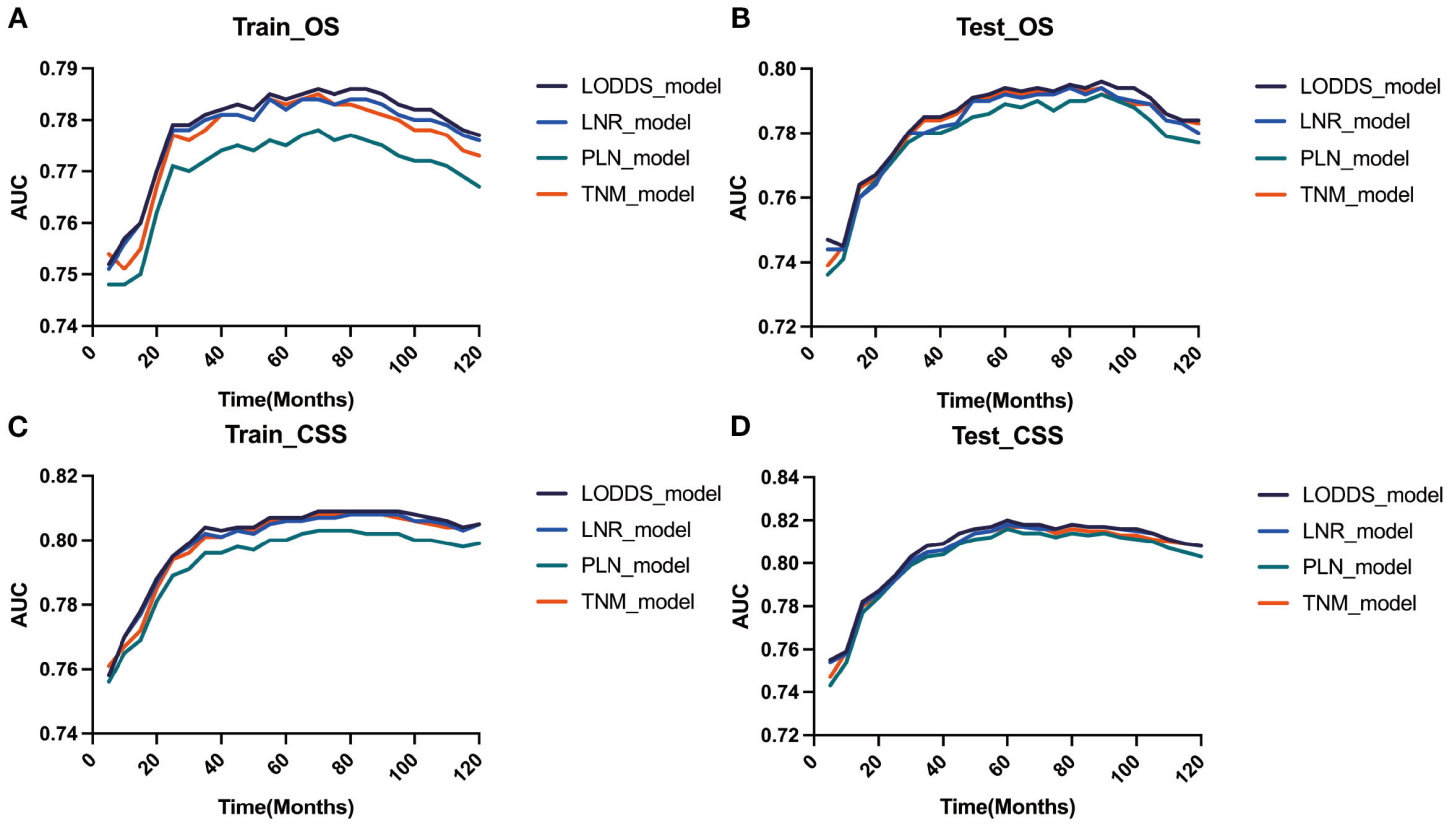
Evaluation of the nomogram for the OS and CSS of patients with LOGA using DCA based on SEER database. DCA for predicting 1-, 3-, 5- and 10-years OS in the training **(A)** and testing **(B)** cohorts. DCA for predicting 1-, 3-, 5- and 10-years CSS in the training **(C)** and testing **(D)** cohorts.

Based on the time-dependent AUC curves, the LODDS-stage model outperformed all individual variables (**Figure 5**) and other staging systems (**Figure 6**) for both OS and CSS in both the training and testing sets. The predictive efficacy of the LODDS staging-based predictive model for OS (**Figures 7A, B**) and CSS (**Figures 7C, D**) at different time points (1, 3, 5, and 10 years) was evaluated using DCA in the training and testing sets. The results showed that the LODDS-stage model was consistently above the All and None curves at most risk thresholds, suggesting its high clinical applicability. In conclusion, the LODDS-stage model shows good predictive benefits at multiple time points, which is expected to provide a reference basis for clinical individualized treatment decision-making.

### Validation of prognostic prediction models with multiple external datasets

**Figure 8:**
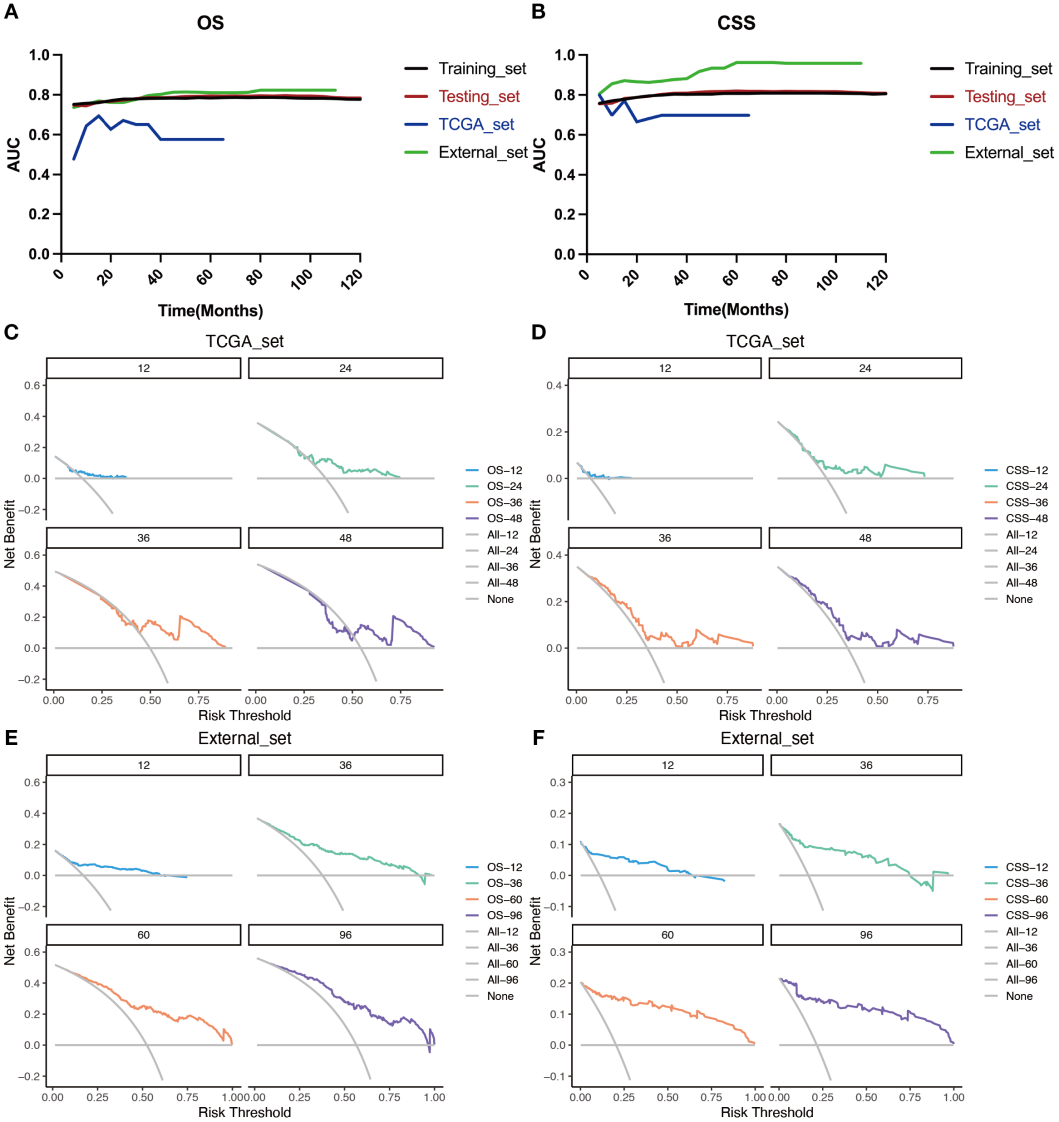
Evaluation of the nomogram for the OS and CSS of patients with LOGA using time‐dependent AUC and DCA based on TCGA database and hospital cohort. Summary of the time‐dependent AUC of the LODDS stage model for the OS **(A)** and CSS **(B)** of four cohorts. DCA for predicting 1-, 2-, 3-, and 4-years OS **(C)** and CSS **(D)** in the TCGA cohort. DCA for predicting 1-, 3-, 5-, and 8-years OS **(E)** and CSS **(F)** in the hospital cohort.

The TCGA dataset and the combined dataset of the two hospitals were used as two external validation cohorts for the constructed predictive models. Both the time-dependent AUC (**Figures 8A, B**) and DCA curves (**Figures 8C-F**) were utilized to demonstrated the accuracy and clinical utility of constructing prediction models based on LODDS-stage.

### Risk classification and survival analysis

**Figure 9:**
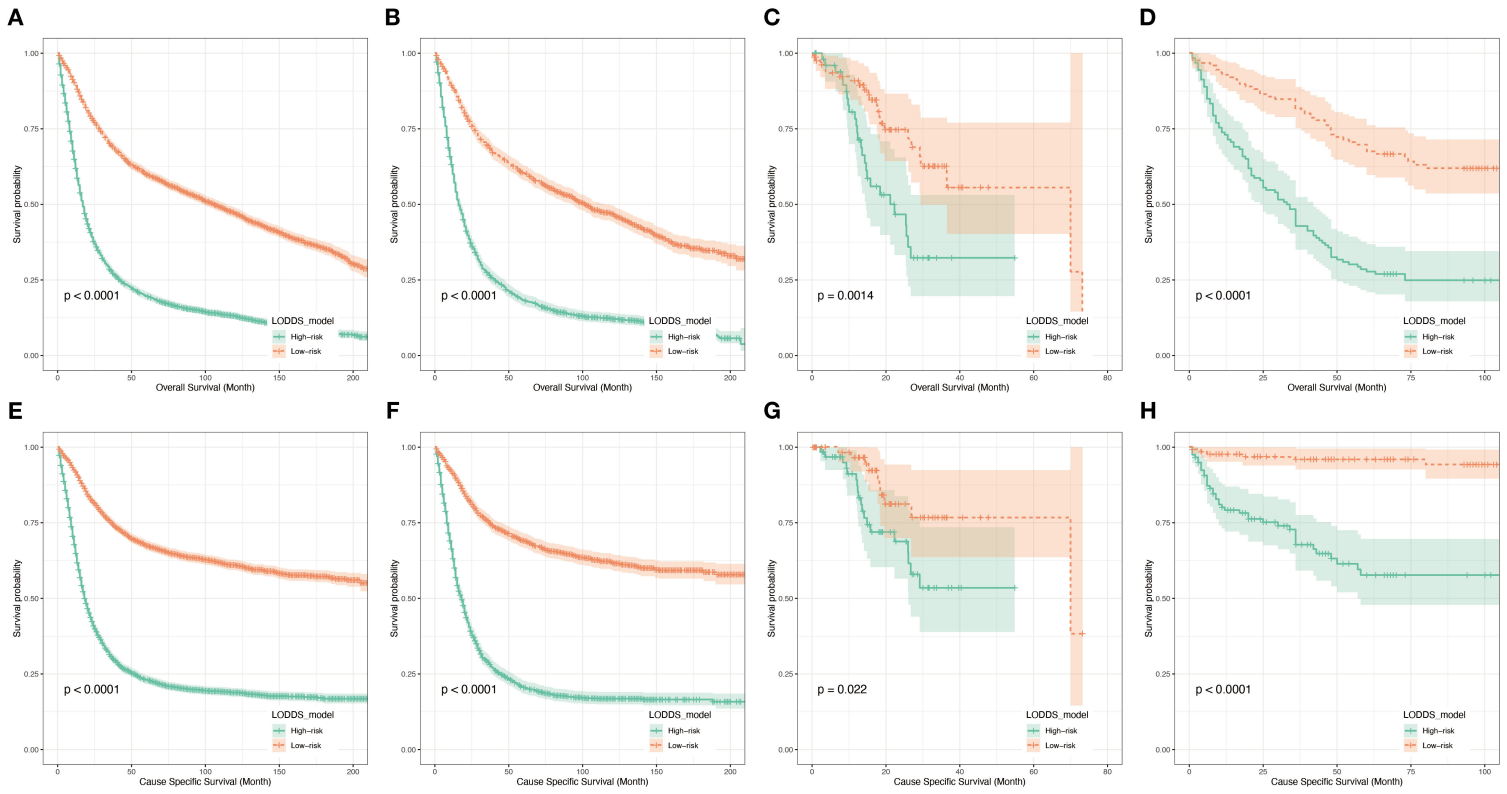
Kaplan-Meier analyses for patients classified based on prognostic risk. K–M curves of OS in the training cohort **(A)**, testing cohort **(B)**, TCGA cohort **(C)** and hospital cohort**(D)**. K–M curves of CSS in the training cohort **(E)**, testing cohort **(F)**, TCGA cohort **(G)** and hospital cohort **(H)**.

To further validate the utility of the prognostic prediction model, patients were equally stratified into two groups based on the median model score: high- and low-risk. Survival analyses were subsequently performed using Kaplan-Meier survival curves for both groups of patients in all datasets(training (**Figures 9A, E**) and testing sets (**Figures 9B, F**) from the SEER database, external validation sets from the TCGA database (**Figures 9C, G**) and dataset consisting of data from two hospitals (**Figures 9D, H**)). The analysis revealed a significantly poorer survival outcomes in the high-risk group compared to the low-risk group, with a statistically significant difference (p < 0.05). Throughout the follow-up period, individuals in the low-risk group consistently exhibited better survival outcomes. These findings suggest that the LODDS model demonstrates the effective discriminatory ability and holds promise as a valuable tool for predicting long-term survival outcomes.

## Discussion

Recent studies have increasingly highlighted the limitations of conventional lymph node staging methods such as N-stage, PLN, and LNR in gastric cancer, particularly in the context of inadequate lymphadenectomy (15). Emerging evidence suggests that LODDS provides a more comprehensive reflection of nodal burden by integrating both positive and negative lymph nodes (16), and several investigations have confirmed its superior prognostic value across different cancer types (13, 17, 18). Nevertheless, most existing studies are retrospective and primarily derived from single-database analyses, which may restrict generalizability. Future research should focus on prospective validation of LODDS-based models in diverse populations, the incorporation of molecular and genomic biomarkers to further enhance predictive accuracy, and the development of user-friendly clinical tools to facilitate real-world application. These directions will not only strengthen the prognostic power of LODDS but also contribute to more precise risk stratification and individualized management in late-onset gastric adenocarcinoma.

Accurate survival prediction is essential for improving personalized treatment and follow-up in patients with LOGA. In this study, we developed a prognostic model that includes age, gender, tumor grade, tumor size, chemotherapy, and LODDS. The model was built using data from the SEER and was validated with patient data from TCGA databases and two hospitals. It showed strong accuracy and good consistency, performing better than traditional systems such as N-stage, PLN, and LNR.

Gender was a significant prognostic factor, with female patients having worse OS in this study. This result differs from previous studies (19, 20) and may be possibly due to hormone changes related to aging. Previous study showed that older female gastric cancer patients exhibit higher rates of poor differentiation and diffuse subtype compared to their male counterparts, despite the less pronounced inter-sex difference (21). Older patients also had poorer outcomes, likely due to diminished physiological reserves and increased vulnerability to treatment-related toxicity (22, 23).

Tumor grade, reflecting the degree of histological differentiation, showed strong prognostic relevance, which was consistent with previous studies (24). In contrast to tumors such as breast (25), lung (26), and liver cancers (27), tumor size is not used in the assessment of T-stage of gastric cancer. But tumor size reflects the proliferative state of the tumor (28) and should be closely related to the patient’s prognosis (29). Our results revealed that larger tumor was linked to worse survival, emphasizing its clinical relevance beyond conventional staging. Thus, tumor size may serve as a supplementary indicator of tumor aggressiveness and progression risk and should not be ignored.

Besides, the model included a binary indicator of whether the patient underwent chemotherapy. Our findings confirmed that patients who received chemotherapy lived longer than those who did not, highlighting the prognostic and therapeutic value of systemic treatment in LOGA.

A notable innovation of this study lies in the incorporation of LODDS. In comparison to N-stage and PLN, LODDS takes into account the extent of lymph node clearance. Compared to LNR, LODDS avoids incorrect assessment of the extent of lymph node metastasis at extreme events and provides a more stable and nuanced assessment of nodal involvement (10). In our study, LODDS consistently outperformed PLN, LNR, and N-stage in stratifying survival risk, particularly in patients with inadequate lymph node retrieval or minimal nodal involvement. This robustness suggests that LODDS is less susceptible to surgical and pathological variability, making it a more reliable parameter in real-world clinical settings.

Another key advantage of this study is the use of external validation from multiple centers, which supports the model’s reliability across different patient groups and clinical settings (30). The SEER database provided reliable, large population-based data. The TCGA database further complemented SEER. The external hospital cohort exemplified the value of the model for real-world clinical applications. This combination enhanced the credibility of the model and demonstrated its potential for practical application in a variety of healthcare settings.

Nevertheless, some limitations should be acknowledged. Some clinically relevant factors, such as lymphadenectomy extent, margin status, and molecular subtype, were not available in the public databases, which may restrict the full predictive capacity of the model. The external hospital dataset contained a smaller number of cases with a shorter follow-up period. A larger number of cases will be added in future studies and these patients will continue to be closely followed up. This study did not adjust for multiple comparisons. therefore, some findings may be at risk of false positives and require validation in larger future studies.

## Conclusions

In conclusion, we developed a robust and clinically applicable prognostic model for LOGA patients by combining key demographic and pathological variables with LODDS—a novel and powerful indicator of nodal burden. The model demonstrated superior predictive performance compared to traditional nodal staging methods and holds promise for risk stratification, postoperative management, and individualized treatment planning in late-onset gastric adenocarcinoma.

## Data Availability

The original contributions presented in the study are included in the article/supplementary material. Further inquiries can be directed to the corresponding author.
